# Effect of the transition from more than adequate iodine to adequate iodine on national changes in the prevalence of thyroid disorders: repeat national cross-sectional surveys in China

**DOI:** 10.1530/EJE-21-0975

**Published:** 2021-11-11

**Authors:** Yongze Li, Zhongyan Shan, Weiping Teng

**Affiliations:** 1 Department of Endocrinology and Metabolism, The Institute of Endocrinology, NHC Key Laboratory of Diagnosis and Treatment of Thyroid Diseases, The First Affiliated Hospital of China Medical University, Shenyang, Liaoning, China

## Abstract

**Objective:**

Longitudinal studies have investigated the effects of changing iodine status on thyroid disorders, but the effect of a transition from more than adequate iodine to adequate iodine on national changes in prevalence adjusted for changing risk factors remains unclear.

**Design:**

Two repeat nationwide surveys were conducted from 2009–2010 to 2015–2017 to assess changes in thyroid disorder prevalence and iodine status in China.

**Methods:**

A multistage stratified random sampling method was used to obtain a nationally representative sample of urban adults aged 18 and older in mainland China in 2009 (*n* = 14 925) and 2015 (*n* = 12 553). Changes in thyroid disorder prevalence, urinary iodine concentration (UIC), and thyroid-stimulating hormone (TSH) levels were assessed. Logistic regression models were used to examine changes in prevalence over time.

**Results:**

The median UIC decreased significantly from 219.7 to 175.9 μg/L (*P* < 0.0001). The weighted prevalence of overt hyperthyroidism, subclinical hyperthyroidism, Graves’ disease, and goitre decreased between 2009 and 2015 in the overall population (*P* < 0.05 for all). Despite no significant changes in subclinical hyperthyroidism or hypothyroidism or anti-thyroid peroxidase or anti-thyroglobulin antibody positivity prevalence, a significant increase in thyroid nodule prevalence (*P* < 0.0001) was found in the overall population. The 2.5th TSH percentile increased by 0.15 mIU/L (95% CI: 0.01 to 0.30 mIU/L, *P* = 0.04) from 2009 to 2015.

**Conclusions:**

With the iodine status transition from more than adequate to adequate, thyroid disorder (except for thyroid nodules) prevalence remained stable or even decreased after adjusting for confounding factors among adults in mainland China between 2009 and 2015. Additional studies are needed to explore the reasons for the increased thyroid nodule prevalence.

## Introduction

China was considered a region with iodine deficiency prior to 1970 (1). Mandatory universal salt iodization (USI) was thus introduced nationwide in 1996 (2). Over the course of two decades of USI implementation and revision, the Chinese population consecutively experienced an iodine nutrition status of excessive iodine (EI) intake for 5 years, more than adequate iodine intake (MAI) for 10 years, and adequate iodine (AI) intake for 5 years (Supplementary Table 1, see section on supplementary materials given at the end of this article) (3, 4, 5, 6, 7, 8, 9).

To observe the benefits of iodine fortification on the population and identify unexpected negative effects in the early stage, it was important to monitor the USI programme. Ideally, iodine deficiency disorder (IDD) prevention leads to a decline in the prevalence of IDD but not an increase in hypothyroidism or autoimmune thyroid diseases (10). As a moderate increase in iodine intake may cause an increase in the prevalence of hypothyroidism, the USI programme should be introduced cautiously (10).

The main consequence of chronic iodine deficiency in adults is the high prevalence of hyperthyroidism, thyroid nodules, and goitre. Data from the first national survey in 2009, which started a few years after the revision of the USI programme in 2002 and reduced the national standards for iodized salt, revealed a lower prevalence of goitre than in 1999 and an MAI status in the overall population (4, 7). In a 5-year prospective Chinese study, an MAI status was found to lead to hypothyroidism and autoimmune thyroiditis (10). Furthermore, in a cross-sectional population study of adults in China that compared area with AI, the prevalences of subclinical hypothyroidism and positive anti-thyroid antibodies were significantly higher in the area with MAI (11). However, whether a further decrease in the concentration of iodine in salt is sufficient to cause a decrease in the prevalence of IDD in adults is unclear.

The improved iodine supply in China is mirrored by findings from the second national survey in 2015, which demonstrated an AI status in mainland China (5). A previous study indicated that a slight reduction in urinary iodine concentration (UIC) within the AI range was associated with decreased prevalence of IDD and a stable prevalence of markers of autoimmune thyroid disorders (12). Nevertheless, there is a lack of evidence of national changes in the prevalence of thyroid disorders with the transition from MAI to AI status. In addition, dynamic changes in population structure, socioeconomic status, and lifestyles should be taken into consideration when assessing changes in prevalence over time.

To obtain a more accurate and comprehensive understanding of the changes in the prevalence of thyroid disorders in mainland China with the transition from MAI to AI status between 2009 and 2015, this analysis presents nationally representative data from two population-based repeat cross-sectional surveys. Furthermore, we determined temporal changes in thyroid-stimulating hormone (TSH) levels against this background.

## Subjects and methods

### Study population and survey design

The first national cross-sectional study (Epidemiological Survey of Thyroid Disorders in China) was carried out in 2009–2010 to evaluate the prevalence of thyroid disorders and iodine status in the adult population of mainland China. Details of the study design are presented elsewhere (4). In brief, a multistage stratified random sampling method was used to select a nationally representative sample of the urban population aged 18 years or older in China (Supplementary Figure). A further cross-sectional survey (thyroid disorders, iodine status, and diabetes epidemiological survey) was carried out in 2015–2017. We previously described the study design in detail, and a detailed flowchart of the study design can also be found in Supplementary Figure (5, 13, 14). Briefly, a multistage stratified random sampling method was applied for all seven geographical areas to obtain nationally representative samples (Supplementary Figure). For each survey, cities were selected based on geographical location, population size, and economic development status. In addition, districts and residential communities were randomly selected. Further details of the study design are presented in Supplementary Figure. The sampling procedure for both surveys sought to take into account the structure of the urban population in mainland China. The inclusion criteria of this study were as follows: age 18 or older, living in the selected community for at least 5 years, and not pregnant. Ultimately, 14 925 participants in 2009 and 12 553 in 2015 remained eligible for the analysis after the exclusion of participants with missing information regarding sex, age, thyroid function, and thyroid ultrasonography (Supplementary Figure). The basic characteristics of the participants included in and excluded from this study are presented in Supplementary Table 2; this information demonstrates the similar age and sex distributions of the participant groups. The research protocols were approved by the medical ethics committee of China Medical University (serial number: IRB[2008]34 and IRB[2013]115). All the participants provided written informed consent after receiving a thorough explanation of the research procedures.

### Measurements

For each participant, a trained interviewer used a detailed questionnaire to collect information about demographic variables, behavioural factors, and personal medical history. An identical protocol was used to measure body weight and height between 2009 and 2015. Body weight and height were measured according to the 3rd edition of Cardiovascular Survey Methods from the WHO. BMI was calculated by dividing body weight in kilograms by the square of height in metres.

In both surveys, thyroid function was examined using the same reagents and instruments by the same laboratory staff. Serum TSH, thyroid peroxidase antibodies (TPOAb), and thyroglobulin antibodies (TgAb) were measured using an electrochemiluminescence immunoassay with a Cobas 601 analyser (Roche Diagnostic) in the central laboratory in Shenyang. Free thyroxine (fT4) and free triiodothyronine (fT3) levels were measured only if the TSH level was outside the reference range. TSH receptor antibodies (TRAbs) were assessed in subjects with TSH below 0.27 mIU/L. The reference ranges for TSH, fT4, fT3, TPOAb, TgAb, and TRAb were 0.27–4.2 mIU/L, 12.0–22.0 pmol/L, 3.1–6.8 pmol/L, <34.0 IU/mL, <115.0 IU/mL, and ≤1.75 IU/L, respectively, as provided by the test kit manufacturers.

In the first study, the physicians who had received centralized training performed all the thyroid ultrasonography evaluations using a portable instrument (LOGIQ a50, 7.5 MHz; GE Healthcare). UIC in all the participants was determined by the ammonium persulfate method based on the Sandell–Kolthoff (S-K) reaction. The certified reference materials (GBW09108 and GBW09110) were purchased from Guobiao Testing & Certification Co., Ltd. (Beijing, China), and their target values were 70.8 and 224 μg/L, respectively. The intraassay coefficients of variation were 2.5 and 2.6% for GBW09108 and GBW09110, respectively, and the corresponding intraassay coefficients of variance were 3.0 and 2.8%, respectively. In the second study, thyroid ultrasonography was performed by qualified physicians trained by the same trainer at the project centre using the same ultrasonic frequency instrument (LOGIQ 100 PRO, 7.5 MHz; GE Healthcare). All the participants underwent thyroid ultrasonography by qualified sonographers, who had trained and passed an examination at the project centre. Kappa statistics were used for the assessment of agreement between two sonographers when diagnosing thyroid nodules. The kappa statistic and agreement rates were 0.82 and 95%, respectively, which indicated good agreement between the sonographers. Intraclass correlation (ICC) was used to compare the volume of the thyroid gland measured by specialized sonographers at the central laboratory and at the survey site. The findings suggested excellent agreement between the two (ICC = 0.83, *P* < 0.0001). UIC was determined using inductively coupled plasma mass spectrometry (ICP-MS; Agilent 7700x; Agilent Technologies). The certified reference materials (GBW09108, GBW09109, and GBW09110) were purchased from Guobiao Testing & Certification Co., Ltd., and their target values were 70.8, 143, and 224 μg/L, respectively. The intraassay coefficients of variation were 2.3, 1.4, and 2.3% for GBW09108, GBW09109, and GBW09110, respectively, and the corresponding intraassay coefficients of variance were 2.7, 2.5, and 2.4%, respectively. In addition, we previously developed a linear model to transform UIC values measured by the S-K method into values associated with the ICP-MS method as follows (15): UIC_ICP-MS_ = 1.05 × UIC_S-K_ + 0.22.

### Diagnostic criteria

The diagnostic criteria for thyroid disorders, deficient iodine (DI), AI, MAI, and EI are listed in Supplementary Table 3. The reference population was defined by the National Academy of Clinical Biochemistry in the United States as follows: (i) individuals with no detectable thyroid autoantibodies, TPOAb, or TgAb; (ii) individuals with no personal or family history of thyroid dysfunction; (iii) individuals with no visible or palpable goitre; and (iv) individuals who did not receive any medications except oestrogen (16).

### Statistical analysis

An identical statistical plan was used to account for the complex sampling design of the two studies; we used SUDAAN software to obtain estimates of prevalence and s.e. according to the Taylor linearization method. Estimates were weighted to reflect the age, sex, and distribution of geographic regions of adults living in China. Weighting coefficients were derived from 2010 Chinese population census data, and the sampling scheme of the two surveys was aimed to obtain a national estimate. Briefly, the weighting coefficient was the inverse of the adjusted probability of obtaining the data for a respondent; each individual case in the analysis was assigned a certain coefficient (individual weight), by which it was multiplied to represent the actual population with the same characteristics of sex, age, and region. s.e. were calculated with appropriate statistical techniques using data from the complex survey design. To counteract the effect of the changing population structure from 2009 to 2015, age- and sex-specific adjustments were performed using direct standardization, with the standard being all adults across the entire period. Age- and sex-specific standardized coefficients were based on 2010 Chinese population census data. Categorical data were analysed by the *χ*^2^ test or Fisher’s exact test, as appropriate, and the results are presented as percentages and 95% CIs. Continuous data are described with means and 95% CIs or medians and interquartile ranges (IQRs), as appropriate. Logistic regression models were used to examine changes in the prevalence of thyroid disorders between 2009 and 2015. Interactions between two surveys and potential confounders were tested by the addition of the cross-product terms in the logistic regression model, and subsequent stratification of the risk factors was performed if any of those were statistically significant. Multivariate quantile regression analysis was applied to estimate changes in TSH at the 2.5th, 50th, and 97.5th percentiles between the two studies. Statistical significance was defined by a two-sided *P* value <0.05. All statistical analyses were conducted using the SAS system, version 9.3 (SAS Institute Inc, Cary, NC) and SUDAAN software, version 10.0 (Research Triangle Institute).

## Results

### Characteristics of the study participants

Table 1 presents the characteristics of the respondents in each survey. Significant differences were observed in education level, mean BMI, consumption of iodized salt, current cigarette smoking, and family history of thyroid disorders between 2009 and 2015. The median UIC at the time of the survey was significantly decreased from 219.7 (IQR: 148.4 to 304.2 μg/L) to 175.9 μg/L (IQR: 121.3 to 262.2 μg/L). Additionally, the proportion of iodized salt consumption decreased significantly from 98.7 (95% CI: 98.4 to 98.9%) to 94.5% (95% CI: 94.0 to 95.1%), whereas the prevalence of ID increased significantly from 11.3 (95% CI: 10.2 to 12.4%) to 16.7% (95% CI: 15.4 to 18.2%).

**Table 1:** Weighted sample characteristics by survey wave.

Characteristics	2009–2010 survey	2015–2017 survey	*P* of difference
*n*	14 925	12 553	
Mean age at survey	39	38	0.13
Sex			
Men	52.2	51.6	0.8
Women	47.8	48.5	
Consumption of iodized salt	98.7	94.5	<0.0001
Current cigarette smoker	29.3	24.6	0.03
Family history of thyroid disorders	4.8	6.4	0.001
Education			
Less than high school	27.6	21.8	0.0003
High school and above	72.4	78.2	
Mean BMI	23.7	23.4	0.04
Overweight	30.2	26.5	0.03
Obesity	4.9	5.1	0.65
Median urinary iodine concentration (IQR)	219.7 (148.4–304.2)	175.9 (121.3–262.2)	<0.0001
Iodine status			
Deficient iodine	11.3	16.7	<0.0001
Adequate iodine	31.1	43.7	<0.0001
More than adequate iodine	31.9	19.9	<0.0001
Excessive iodine	25.7	19.7	0.003

### Changes in prevalence of thyroid disorders

Table 2 presents the changes in the weighted prevalence of thyroid disorders in mainland China. After adjusting for confounding factors, the prevalence of overt hyperthyroidism (0.7% (95% CI: 0.6 to 0.9%) vs 0.5% (95% CI: 0.4 to 0.6%), *P* = 0.02), subclinical hyperthyroidism (0.5% (95% CI: 0.4 to 0.6%) vs 0.3% (95% CI: 0.3 % to 0.5%), *P* = 0.04), Graves’ disease (0.5% (95% CI: 0.4 to 0.7%) vs 0.3% (95% CI: 0.2 to 0.4%), *P* = 0.006), and goitre (1.6% (95% CI: 1.4 to 1.8%) vs 0.6% (95% CI: 0.5 to 0.8%), *P* < 0.0001) decreased significantly between 2009 and 2015 in the overall population.

**Table 2:** Changes in the weighted prevalence of thyroid disorders between 2009 and 2015 among adults in China. The odds ratio was adjusted for BMI, family history of thyroid disorders, education level, and smoking status.

Thyroid disorders	Prevalence (95% CI)		Adjusted OR (95% CI)	*P*-value
	2009–2010	2015–2017		
*n*	14 925	12 553		
Overt hyperthyroidism	0.7 (0.6–0.9)	0.5 (0.4–0.6)	0.66 (0.47–0.95)	0.02
Subclinical hyperthyroidism	0.5 (0.4–0.6)	0.3 (0.3–0.5)	0.65 (0.42–0.99)	0.04
Graves’ disease	0.5 (0.4–0.7)	0.3 (0.2–0.4)	0.57 (0.38–0.85)	0.006
Overt hypothyroidism	0.8 (0.7–1.0)	1.6 (0.5–5.0)	1.73 (0.58–5.13)	0.33
Subclinical hypothyroidism	14.9 (12.9–17.2)	15.6 (13.2–18.4)	0.93 (0.72–1.20)	0.57
Positive TPOAb	8.9 (8.2–9.7)	10.9 (8.3–14.3)	1.18 (0.88–1.59)	0.26
Positive TgAb	9.6 (8.7–10.6)	9.1 (7.5–11.1)	0.87 (0.70–1.09)	0.23
Goitre	1.6 (1.4–1.8)	0.6 (0.5–0.8)	0.32 (0.24–0.42)	<0.0001
Thyroid nodule	13.4 (12.4–14.5)	22.5 (20.7–24.3)	1.66 (1.46–1.88)	<0.0001

In contrast, no significant change in the prevalence of overt hypothyroidism (0.8% (95% CI: 0.7 to 1.0%) vs 1.6% (95% CI: 0.5 to 5.0%), *P* = 0.33), subclinical hypothyroidism (14.9% (95% CI: 12.9 to 17.2%) vs 15.6% (95% CI: 13.2 to 18.4%), *P* = 0.57), TPOAb positivity (8.9% (95% CI: 8.2 to 9.7%) vs 10.9% (95% CI: 8.3 to 14.3%), *P* = 0.26), or TgAb positivity (9.6% (95% CI: 8.7 to 10.6%) vs 9.1% (95% CI: 7.5 to 11.1%), *P* = 0.23) was observed in the overall population.

The prevalence of thyroid nodules increased significantly from 13.4 (95% CI: 12.4 to 14.5%) to 22.5% (95% CI: 20.7 to 24.3%) in 2009–2015.

### Changes in TSH at the 2.5th, 50th, and 97.5th percentiles

Table 3 illustrates the changes in TSH levels at the 2.5th, 50th, and 97.5th percentiles between the first and second studies in the overall and reference populations, adjusted for age, sex, BMI, family history of thyroid disorders, education level, and smoking status. The 2.5th percentile of TSH significantly increased by 0.15 mIU/L (95% CI: 0.01 to 0.30 mIU/L, *P* = 0.04) and 0.13 mIU/L (95% CI: 0.01 to 0.26 mIU/L, *P* = 0.04) in the overall and reference populations, respectively. However, no significant changes in TSH at the 50th and 97.5th percentiles were observed in the overall and reference populations.

**Table 3:** Changes in serum TSH levels at the 2.5th, 50th, and 97.5th percentiles between 2009 and 2015 among adults in China. The changes in quantiles of TSH were adjusted for BMI, family history of thyroid disorders, education level, and smoking status.

TSH percentile	TSH levels (95% CI)		Adjusted change in quantiles of TSH (95% CI)	*P*-value
	2009–2010	2015–2017		
Overall population				
*n*	14 925	12 553		
2.5th	0.66 (0.61 to 0.70)	0.73 (0.69 to 0.79)	0.15 (0.01 to 0.30)	0.04
50th	2.36 (2.28 to 2.48)	2.43 (2.34 to 2.54)	−0.003 (−0.14 to 0.14)	0.96
97.5th	7.88 (7.53 to 8.25)	7.70 (7.21 to 8.61)	−0.10 (−1.47 to 1.26)	0.88
Reference population				
*n*	11 489	9879		
2.5th	0.78 (0.75 to 0.82)	0.80 (0.76 to 0.86)	0.13 (0.01 to 0.26)	0.04
50th	2.32 (2.25 to 2.44)	2.42 (2.33 to 2.53)	−0.003 (−0.15 to 0.14)	0.96
97.5th	6.71 (6.49 to 6.97)	7.21 (6.15 to 8.45)	0.20 (−0.87 to 1.26)	0.72

### Subgroup and sensitive analyses

Subgroup and sensitivity analyses for changes in thyroid disorder prevalence are provided in the Supplement. Overall, changes in the prevalence of overt hyperthyroidism, subclinical hyperthyroidism, Graves’ disease, overt hypothyroidism, subclinical hypothyroidism, TPOAb positivity, TgAb positivity, goitre, and thyroid nodules remained stable according to three logistic regression models with progressively increased adjustment of risk factors in the overall population (Supplementary Table 4). Results of stratification of the risk factors are presented in Supplementary Tables 5, 6, 7, and 8. In addition, we validated the ICP-MS method to calibrate the S-K method by using the previously developed equation. We calculated the median UIC of 231.0 μg/L (95% CI: 223.0 to 240.7 μg/L) among the overall population in: 2009–2010, which was significantly higher than the median UIC of 175.9 μg/L (95% CI: 169.5 to 184.0 μg/L) in 2015–2017 (*P* < 0.0001).

## Discussion

In this study, we investigated the effects of the transition from MAI to AI on national changes in the prevalence of thyroid disorders between 2009 and 2015 (13 and 19 years after the initiation of the USI programme, respectively) based on national representative data from mainland China.

We demonstrate a decrease in the prevalence of IDD, such as hyperthyroidism and goitre, as well as an increase in serum TSH levels at the 2.5th percentile. However, the prevalence of thyroid nodules increased, and this could not be explained by the improved thyroid ultrasound device because the same resolution (7.5 MHz) was used to evaluate thyroid nodules with the same diagnostic criteria (>5 mm in diameter). Our data, therefore, suggest a decline in IDD during recent decades, even though the prevalence of thyroid disorders related to iodine repletion, such as hypothyroidism and thyroid antibody positivity, was stable during that time period.

Iodine intake has been found to greatly affect thyroid disorders in the population (4, 5). Even small changes in the level of iodine intake in a population can alter the risk of thyroid-related diseases (17). We found that the median UIC decreased from 2009 to 2015. This decrease may be explained by changes in the salt iodization policy in 2012 (3). Thus, the iodine status changed from MAI to AI, accompanied by a decreased rate of iodized salt consumption during that time period.

When the iodine supply increases, there is a risk of overcompensation, which may lead to the occurrence of autoimmune thyroid disorders. Indeed, sudden and excessive increases in iodine intake may cause the formation of thyroid antibodies (10). In this study, we observed a stable prevalence of thyroid antibody positivity that paralleled the tendency of optimized iodine status between the first and second surveys. Previous studies of trends have focused mainly on the impact of sudden or periodic increases in iodine supply in the population on thyroid autoimmunity (18, 19). In contrast, we examined the effect of the transition from MAI to AI status on the same outcome. In our previous study conducted 4 years after the initiation of the USI programme, no difference in the 5-year incidence rate of thyroid autoimmunity was detected (10). Nonetheless, a Danish study showed that the incidence of thyroid autoimmunity increased after 4–5 years of forced iodine fortification, although this increase was due to increased iodine intake in the population from previous moderate and mild to mandatory intake (18). Similarly, a study in Slovenia found that after 10 years of increased iodine fortification, the iodine status changed from mild deficiency to sufficient, and the incidence of TPOAb positivity significantly increased (19).

The current and previous iodine supply in a region determines the distribution of TSH levels in the population, and the TSH reference interval in a region with iodine deficiency is often lower than that in a region with a higher iodine supply (20, 21). However, when a region shifts from iodine deficiency to sufficient iodine, the distribution of TSH may initially shift to the left (22). With continuous improvement in the iodine supply over several years, the distribution of TSH levels for the general population shifts to the right (23). Even the TSH reference range established during the transition from deficiency to sufficiency may not reflect the current situation (23). In our study, the TSH level at the 50th and 97.5th percentiles did not change significantly between 2009–2010 and 2015–2017 after adjusting for confounding factors. This was not consistent with a previous study reporting an increased median TSH with a slightly decreasing median UIC in Northeast Germany (12). Nevertheless, we found that the 2.5th percentile of serum TSH levels increased significantly in the overall and reference populations, which resulted in a decreased prevalence of hyperthyroidism. We assume that the change in TSH was mainly related to the improved iodine supply. A previous study indicated that an increase in UIC was significantly associated with an increase in the 50th and 97.5th percentiles but a decrease in the 2.5th percentile of serum TSH (24). In contrast, the increase in TSH at the 2.5th percentile might be related to the decrease in UIC.

Iodine supplementation in a population may lead to a higher prevalence of hypothyroidism (25). Our previous study was conducted in historically iodine-deficient areas after iodine fortification, and according to the results, the prevalence of overt hypothyroidism did not increase, consistent with this study. However, there was a slight increase in subclinical hypothyroidism in that study (10). In contrast, a study from Germany found a decrease in the prevalence of hypothyroidism after decreased iodine intake from 123.0 to 112.0 μg/L (12). Differences in results between our study and other studies might be mainly explained by the different follow-up periods and populations with differences in iodine status before and during the implementation of USI. Hence, the decrease in the prevalence of IDDs and the stable prevalence of hypothyroidism indicates an optimal but slightly declining iodine supply in the Chinese population over the past decade.

The strength of our study is that, to our knowledge, it is the first to describe the change in the prevalence of thyroid disorders and TSH levels in a population with a transition from an MAI to AI status in national surveys. The limitations of the current study also warrant discussion. First, urinary iodine was measured using different methods in the two surveys. However, previous studies have demonstrated that the Sandell–Kolthoff reaction and inductively coupled plasma mass spectrometry methods show good congruence, and the test results of the two methods could be compared directly (15). Secondly, because repeat cross-sectional surveys were involved, this study was not able to reveal the mechanisms involved in the observed phenomena. Thirdly, although the survey staff were highly trained, their efficacy or skill level may have resulted in some misclassification. Fourthly, we analysed data with SUDAAN software for the statistical analysis of correlated data to account for the complex survey design using the weights assigned to the individuals sampled to represent the Chinese population, but rural residents were not included in this study; nevertheless, a previous study indicated that urbanization did not affect iodine status or the prevalence of thyroid disorders in China (5).

In conclusion, the improved iodine supply with the transition of iodine status from MAI to AI between 2009 and 2015 in China was paralleled by a reduction in the prevalence of IDD and an increase in the lower limits of TSH. Furthermore, there was no increase in the prevalence of hypothyroidism and autoimmune thyroid disorders, indicating an optimal iodine supply in the general adult population in China. However, the increase in thyroid nodule prevalence is alarming, and more studies are needed to explore the potential reason for this increase.

## Supplementary Material

Supplementary Figure. Flowchart depicting the survey design. For the sampling process of the first survey in 2009-2010, 10 provinces and autonomous regions were selected from all 7 geographic regions in mainland China. One to three cities were selected from each geographic area in all 7 geographic regions of mainland China. In the second stage, one district was randomly selected from each city. In the third-stage, one or two residential communities were randomly selected from each district. At the final stage, eligible individuals from the local resident registration list who met the inclusion criteria were randomly selected according to age and sex composition among the population from China national census data. For the sampling process of the second survey in 2015-2017, in the first stage, one city was selected from each province in all 31 provinces of mainland China. Ten cities were further selected in accordance with the first survey for the current analysis. In the second stage, one district was randomly selected from each city. In the third stage, two residential communities were randomly selected from each district. At the final stage, eligible individuals from the local resident registration list who met the inclusion criteria were randomly selected according to age and sex composition among the population from China national census data.

Supplementary Table 1. Iodine status in China before and after the introduction of USI

Supplementary Table 2. Unweighted basic characteristics of the participants included in and excluded from this survey

Supplementary Table 3. Diagnostic Criteria for Iodine Status and Thyroid Disorders

Supplementary Table 4. Changes in the weighted prevalence of thyroid disorders between 2009 and 2015 among adults in China

Supplementary Table 5. Changes in the weighted prevalence of thyroid disorders stratified by family history of thyroid disorders between 2009 and 2015 among adults in China

Supplementary Table 6. Changes in the weighted prevalence of thyroid disorders stratified by BMI group between 2009 and 2015 among adults in China

Supplementary Table 7. Changes in the weighted prevalence of thyroid disorders stratified by education level between 2009 and 2015 among adults in China

Supplementary Table 8. Changes in the weighted prevalence of thyroid disorders stratified by smoking status between 2009 and 2015 among adults in China

## Declaration of interest

The authors declare that there is no conflict of interest that could be perceived as prejudicing the impartiality of this study.

## Funding

This work is supported by the National Natural Science Foundation of China http://dx.doi.org/10.13039/501100001809 (Grant No. 81970682 and 82000753), the Key Laboratory Project of Thyroid Diseases, National Health Commission (Grant No. 2019PT33001), the Research Foundation of Innovative Team in Advanced Educational Institute (Grant No. LT201215), China Postdoctoral Science Foundation http://dx.doi.org/10.13039/501100002858 (Grant No. 2021MD703910) and the Research Fund for Public Welfare from the National Health and Family Planning Commission of China (Grant No. 201402005).

## Author contribution statement

Yongze Li, Zhongyan Shan, and Weiping Teng conceived and designed the study. Zhongyan Shan and Weiping Teng supervised the study. Yongze Li performed the statistical analysis. The Thyroid Disorders in China Epidemiological Survey Group and the Thyroid Disorders, Iodine Status, and Diabetes Epidemiological Survey Group conducted the epidemiological survey. All authors contributed to the acquisition, analysis, or interpretation of data. Yongze Li drafted the manuscript. All authors revised the report and approved the final version before submission. Zhongyan Shan and Weiping Teng are the guarantors and attest that all listed authors meet authorship criteria and that no others meeting the criteria have been omitted.
