# Supplementary material for: Effect of the transition from more than adequate iodine to adequate iodine on national changes in the prevalence of thyroid disorders: repeat national cross-sectional surveys in China
Source: Eur J Endocrinol. 2021 Nov 11;186(1):115–22. doi: 10.1530/EJE-21-0975 (PMC8679845; doi:10.1530/EJE-21-0975)
Supplement: Supplementary Figure. Flowchart depicting the survey design. For the sampling process of the first survey in 2009-2010, 10 provinces and autonomous regions were selected from all 7 geographic regions in mainland China. One to three cities were selected from each geographic area in all 7 geographic r [file supplementary_figure_1.pdf]

The first cross-sectional study in 2009-2010

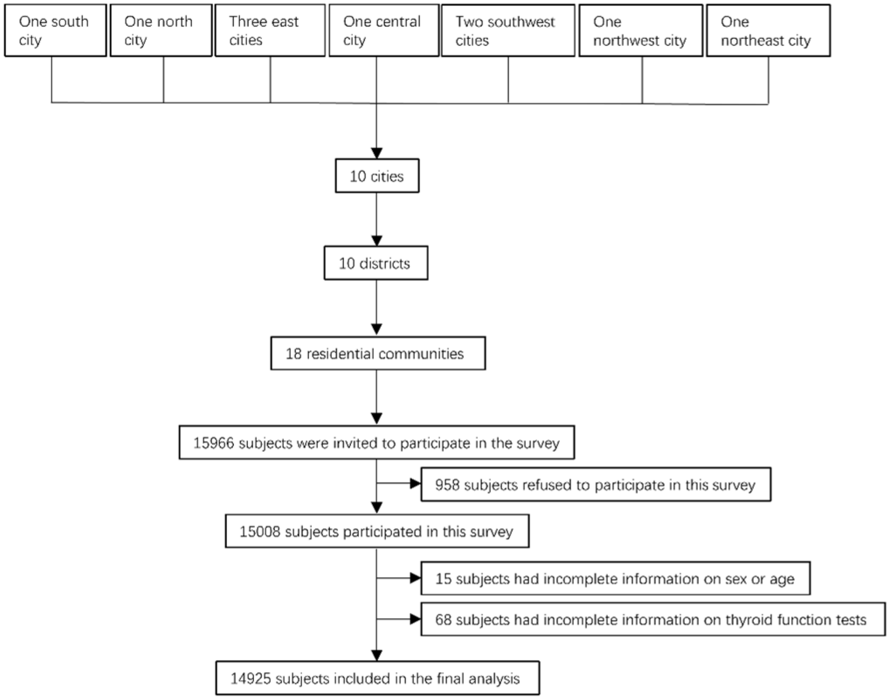

The second cross-sectional study in 2015-2017

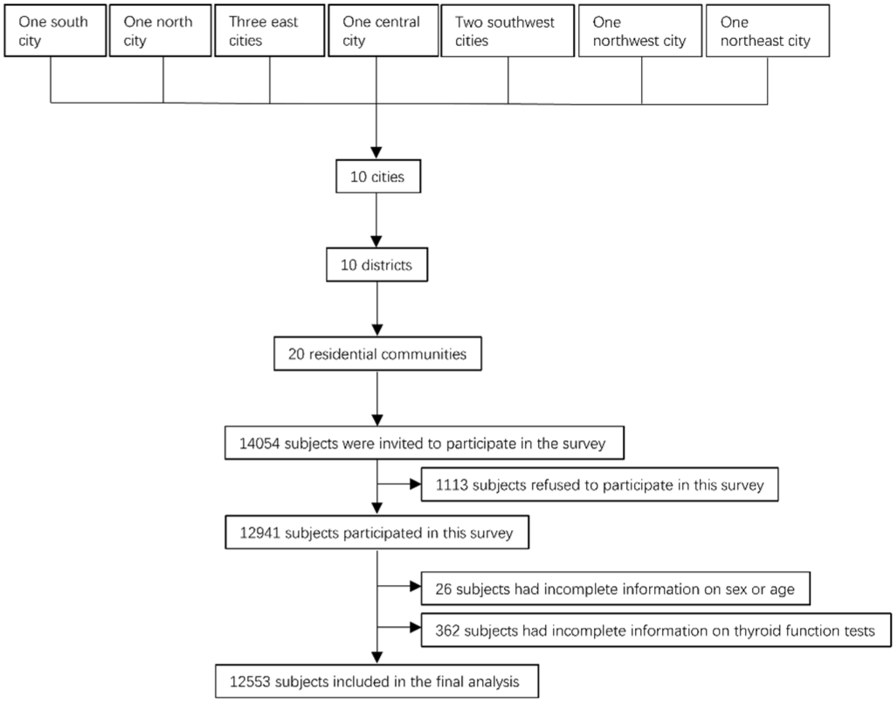

The number of the included subjects in each survey site

	South China			North China			East China									Central China			Southwest China						Northwest China			Northeast China		
Survey phase	City	Site	No.	City	Site	No.	City	Site	No.	City	Site	No.	City	Site	No.	City	Site	No.	City	Site	No.	City	Site	No.	City	Site	No.	City	Site	No.
2009-2010	Guangzhou	Site 1	782	Beijing	Site 1	864	Shanghai	Site 1	831	Nanjing	Site 1	824	Ji'nan	Site 1	796	Wuhan	Site 1	848	Chengdu	Site 1	1350	Guiyang	Site 1	972	Xi'an	Site 1	660	Shenyang	Site 1	1513
		Site 2	719		Site 2	667		Site 2	668		Site 2	714		Site 2	703		Site 2	639					Site 2	537		Site 2	838			
2015-2017	Guangzhou	Site 1	625	Beijing	Site 1	473	Shanghai	Site 1	774	Nanjing	Site 1	561	Ji'nan	Site 1	532	Wuhan	Site 1	695	Chengdu	Site 1	519	Guiyang	Site 1	803	Xi'an	Site 1	521	Dalian	Site 1	746
		Site 2	781		Site 2	622		Site 2	701		Site 2	726		Site 2	334		Site 2	734		Site 2	540		Site 2	523		Site 2	692		Site 2	651
