# Supplementary material for: Effect of the transition from more than adequate iodine to adequate iodine on national changes in the prevalence of thyroid disorders: repeat national cross-sectional surveys in China
Source: Eur J Endocrinol. 2021 Nov 11;186(1):115–22. doi: 10.1530/EJE-21-0975 (PMC8679845; doi:10.1530/EJE-21-0975)
Supplement: Supplementary Table 1. Iodine status in China before and after the introduction of USI [file supplementary_table_1.pdf]

Supplementary Table 1. Iodine status in China before and after the introduction of USI

Year	USI policy	Iodized salt policy	Median UIC in school-age children, ug/L	Iodine levels in household salt, mg/kg	Goitre, %	
					Palpation	Ultrasound
1995	No USI	—	164.8	16.2	20.4	—
1996	USI implemented	The iodine concentration of iodized salt at the production level, retail level and household level was recommended to be 50 mg/kg, 30 mg/kg and 20 mg/kg, respectively	330.2	37	10.9	9.6
1999	USI	—	306.0	42.3	8.8	8.0
2002	USI revised	Reduced the national standards for iodized salt from 50 mg/kg to 35±15 mg/kg at the production level	241.2	31.4	5.8	5.1
2005	USI	—	246.3	—	—	5.0
2012	USI revised	Reduced the iodine concentration in salt to 20-30 mg/kg at the production level, and the provincial government was authorized to set the local iodine concentration in salt to within ±30% of the recommended level based on the local iodine nutritional environment	—	—	—	—

Note: Data were derived from Sun X, et al. Effects of increased iodine intake on thyroid disorders. 2014;29(3):240-7.
