# Supplementary material for: Effect of the transition from more than adequate iodine to adequate iodine on national changes in the prevalence of thyroid disorders: repeat national cross-sectional surveys in China
Source: Eur J Endocrinol. 2021 Nov 11;186(1):115–22. doi: 10.1530/EJE-21-0975 (PMC8679845; doi:10.1530/EJE-21-0975)
Supplement: Supplementary Table 2. Unweighted basic characteristics of the participants included in and excluded from this survey [file supplementary_table_2.pdf]

Supplementary Table 2. Unweighted basic characteristics of the participants included in and excluded from this survey

	Participants		Chi-square or t-value	P value
	Excluded	Included		
2009-2010 Survey	n=958	n=14925		
Age, mean (SD)	45.03 (14.84)	45.49 (14.91)	-0.93	0.35
Men, N (%)	417 (43.5%)	6293 (42.2%)	0.686	0.41
2015-2017 Survey	n=1113	n=12553		
Age, mean (SD)	44.50 (15.14)	43.62 (15.74)	1.854	0.06
Men, N (%)	527 (47.3%)	6049 (48.2%)	0.288	0.59
