# Supplementary material for: Effect of the transition from more than adequate iodine to adequate iodine on national changes in the prevalence of thyroid disorders: repeat national cross-sectional surveys in China
Source: Eur J Endocrinol. 2021 Nov 11;186(1):115–22. doi: 10.1530/EJE-21-0975 (PMC8679845; doi:10.1530/EJE-21-0975)
Supplement: Supplementary Table 3. Diagnostic Criteria for Iodine Status and Thyroid Disorders [file supplementary_table_3.pdf]

**Supplementary Table 3. Diagnostic Criteria for Iodine Status and Thyroid Disorders**

| No. | Disorders | Diagnostic criteria |
|---|---|---|
| 1. | Deficient iodine | UIC <100μg/L |
| 2. | Adequate iodine | UIC 100-199μg/L |
| 3. | More than adequate iodine | UIC 200-299μg/L |
| 4. | Excessive iodine | UIC ≥300μg/L |
| 5. | Overt hyperthyroidism | TSH<0.27mIU/L; FT4>22pmol/L or FT3>6.8pmol/L |
| 6. | Subclinical hyperthyroidism | TSH<0.27mIU/L; FT4 and FT3 within the normal range (FT4 12.0-22.0pmol/L; FT3 3.1-6.8pmol/L) |
| 7. | Graves' disease | Overt hyperthyroidism or subclinical hyperthyroidism; TRAb>1.75IU/L or a diffuse goitre on B-mode ultrasonography |
| 8. | Overt hypothyroidism | TSH>4.2mIU/L; FT4<12pmol/L |
| 9. | Subclinical hypothyroidism | TSH>4.2mIU/L; FT4 within 12-22pmol/L |
| 10. | TPOAb positive | TPOAb>34 IU/ml |
| 11. | TgAb positive | TgAb>115 IU/ml |
| 12. | Goitre | Female >22.5 ml; Male >25.4 ml |
