# Supplementary material for: Effect of the transition from more than adequate iodine to adequate iodine on national changes in the prevalence of thyroid disorders: repeat national cross-sectional surveys in China
Source: Eur J Endocrinol. 2021 Nov 11;186(1):115–22. doi: 10.1530/EJE-21-0975 (PMC8679845; doi:10.1530/EJE-21-0975)
Supplement: Supplementary Table 4. Changes in the weighted prevalence of thyroid disorders between 2009 and 2015 among adults in China [file supplementary_table_4.pdf]

Supplementary Table 4. Changes in the weighted prevalence of thyroid disorders between 2009 and 2015 among adults in China

Thyroid disorder	Model	Odds ratio (95%CI)	P value	P-interaction			
				*family history of thyroid disorders interaction	*BMI group interaction	*education level interaction	*smoking status interaction
Overt hyperthyroidism	1	0.71 (0.52-0.98)	0.04	0.66	0.17	0.59	0.09
	2	0.66 (0.47-0.95)	0.02	0.87	0.12	0.99	0.13
Subclinical hyperthyroidism	1	0.65 (0.42-0.99)	0.04	0.19	0.44	0.04	0.85
	2	0.57 (0.39-0.83)	0.003	0.4	0.74	0.45	0.47
Graves' disease	1	2.02 (0.61-6.64)	0.25	0.02	0.17	0.4	0.09
	2	1.73 (0.58-5.13)	0.33	0.03	0.19	0.5	0.04
Overt hypothyroidism	1	0.93 (0.72-1.20)	0.57	0.18	0.15	0.44	0.7
	2	1.26 (0.91-1.73)	0.16	0.03	0.02	0.1	0.56
Subclinical hypothyroidism	1	0.94 (0.74-1.20)	0.64	0.009	0.04	0.17	0.5
	2	0.87 (0.70-1.09)	0.23	0.05	0.07	0.76	0.69
Positive TPOAb	1	0.32 (0.24-0.42)	<0.0001	0.61	0.95	0.94	0.29
	2	1.87 (1.63-2.14)	<0.0001	0.19	0.004	0.2	0.18
Positive TgAb	1	0.71 (0.52-0.98)	0.04	0.66	0.17	0.59	0.09
	2	0.66 (0.47-0.95)	0.02	0.87	0.12	0.99	0.13
Goitre	1	0.65 (0.42-0.99)	0.04	0.19	0.44	0.04	0.85
	2	0.57 (0.39-0.83)	0.003	0.4	0.74	0.45	0.47
Thyroid nodule	1	2.02 (0.61-6.64)	0.25	0.02	0.17	0.4	0.09
	2	1.73 (0.58-5.13)	0.33	0.03	0.19	0.5	0.04

Model 1: unadjusted model. Model 2: BMI, education level, smoking status, and family history of thyroid disorders. Effect modification was assessed by including interaction terms.
