# Supplementary material for: Effect of the transition from more than adequate iodine to adequate iodine on national changes in the prevalence of thyroid disorders: repeat national cross-sectional surveys in China
Source: Eur J Endocrinol. 2021 Nov 11;186(1):115–22. doi: 10.1530/EJE-21-0975 (PMC8679845; doi:10.1530/EJE-21-0975)
Supplement: Supplementary Table 5. Changes in the weighted prevalence of thyroid disorders stratified by family history of thyroid disorders between 2009 and 2015 among adults in China [file supplementary_table_5.pdf]

Supplementary Table 5. Changes in the weighted prevalence of thyroid disorders stratified by family history of thyroid disorders between 2009 and 2015 among adults in China

		Family history of thyroid disorders			
		Yes		No	
Thyroid disorders	Model	Odds ratio (95%CI)	P value	Odds ratio (95%CI)	P value
Overt hypothyroidism	1	0.37 (0.16-0.90)	0.03	2.28 (0.66-7.84)	0.19
	2	0.39 (0.15-1.00)	0.05	1.95 (0.63-6.03)	0.24
Positive TPOAb	1	0.87 (0.63-1.21)	0.4	1.22 (0.89-1.69)	0.22
	2	0.58 (0.44-0.77)	0.0001	0.97 (0.75-1.26)	0.83
Positive TgAb	1	0.37 (0.16-0.90)	0.03	2.28 (0.66-7.84)	0.19
	2	0.39 (0.15-1.00)	0.05	1.95 (0.63-6.03)	0.24

Model 1: unadjusted model. Model 2: adjusted for BMI, education level, smoking status, and family history of thyroid disorders.
