# Supplementary material for: Effect of the transition from more than adequate iodine to adequate iodine on national changes in the prevalence of thyroid disorders: repeat national cross-sectional surveys in China
Source: Eur J Endocrinol. 2021 Nov 11;186(1):115–22. doi: 10.1530/EJE-21-0975 (PMC8679845; doi:10.1530/EJE-21-0975)
Supplement: Supplementary Table 6. Changes in the weighted prevalence of thyroid disorders stratified by BMI group between 2009 and 2015 among adults in China [file supplementary_table_6.pdf]

Supplementary Table 6. Changes in the weighted prevalence of thyroid disorders stratified by BMI group between 2009 and 2015 among adults in China

		BMI group					
		<25		25-<30		≥30	
Thyroid disorders	Model	Odds ratio (95%CI)	P value	Odds ratio (95%CI)	P value	Odds ratio (95%CI)	P value
Positive TPOAb	1	1.34 (0.87-2.05)	0.18	0.86 (0.70-1.07)	0.17	1.64 (1.02-2.63)	0.04
	2	1.30 (0.87-1.95)	0.2	0.86 (0.70-1.06)	0.16	1.61 (1.01-2.56)	0.04
Positive TgAb	1	0.92 (0.67-1.26)	0.6	0.71 (0.58-0.89)	0.002	1.27 (0.81-2.00)	0.3
	2	1.90 (1.58-2.27)	<0.0001	1.34 (1.10-1.63)	0.003	1.06 (0.74-1.52)	0.76
Thyroid nodule	1	1.34 (0.87-2.05)	0.18	0.86 (0.70-1.07)	0.17	1.64 (1.02-2.63)	0.04
	2	1.30 (0.87-1.95)	0.2	0.86 (0.70-1.06)	0.16	1.61 (1.01-2.56)	0.04

Model 1: unadjusted model. Model 2: adjusted for BMI, education level, smoking status, and family history of thyroid disorders.
