# Supplementary material for: Effect of the transition from more than adequate iodine to adequate iodine on national changes in the prevalence of thyroid disorders: repeat national cross-sectional surveys in China
Source: Eur J Endocrinol. 2021 Nov 11;186(1):115–22. doi: 10.1530/EJE-21-0975 (PMC8679845; doi:10.1530/EJE-21-0975)
Supplement: Supplementary Table 8. Changes in the weighted prevalence of thyroid disorders stratified by smoking status between 2009 and 2015 among adults in China [file supplementary_table_8.pdf]

Supplementary Table 8. Changes in the weighted prevalence of thyroid disorders stratified by smoking status between 2009 and 2015 among adults in China

		Smoking status			
		Current non-smoker		Current smoker	
Thyroid disorders	Model	Odds ratio (95%CI)	P value	Odds ratio (95%CI)	P value
Overt hypothyroidism	1	2.19 (0.62-7.69)	0.22	0.60 (0.27-1.32)	0.2
	2	2.09 (0.66-6.60)	0.21	0.47 (0.19-1.18)	0.11

Model 1: unadjusted model. Model 2: adjusted for BMI, education level, smoking status, and family history of thyroid disorders.
